# Finding One's Footing When Everyone Has an Opinion. Negotiating an Acceptable Identity After Sexual Assault

**DOI:** 10.3389/fpsyg.2021.649530

**Published:** 2021-07-01

**Authors:** Ingrid Dundas, Elin Mæhle, Signe Hjelen Stige

**Affiliations:** ^1^Institute for Clinical Psychology, University of Bergen, Bergen, Norway; ^2^Centre for Crisis Psychology, University of Bergen, Bergen, Norway

**Keywords:** qualitative, sexual assault, rape, identity, acceptable, self-representation, trauma, disclosure

## Abstract

Identities used to describe oneself after trauma may influence recovery, and searches for acceptable identities after sexual assault can be challenging. Fifteen Norwegian female survivors of sexual assault were recruited at a clinical center, and were individually interviewed about post-assault discussions with others. Our focus was on the experiences of non-blaming and believing interactions with others, and how these interactions can be understood as a process of searching for acceptable identities after sexual assault. A reflexive thematic analysis resulted in four themes: *navigating between other people's stories and one's own*; *realizing the seriousness of the assault without drowning in the upset of others*; *finding a place between too much closeness and too much distance*; and *being more than a victim*. We discuss the importance of participants retaining agency in post-assault interactions. We suggest that being a survivor of sexual assault increases the probability, even in believing and non-blaming interactions, of being cast in a subject–object relationship with less freedom and agency than before. Navigating toward acceptable identities may mean working one's way back to being a subject in a subject–subject relationship again.

## Introduction

Identities are formed through interactions. From infancy and onwards, meaning making, including meaning about oneself, is a process that takes place in interaction with others (e.g., Trevarthen, 2001). Meanings may be based on actions and non-verbal interaction as well as verbal exchanges, and some may be implicit rather than conscious. As we develop language, we develop meaning through inner dialogue as well. Through these outer and inner interactions, we develop our identities, our stories about ourselves. Potential beliefs about oneself, including what one thinks, feels, and wants, are developed through interactions, not only in childhood but during one's entire life. In Stolorow's words, identities are influenced and formed through “reciprocally interacting worlds of experiences” throughout life (Stolorow, 1997, p. 399).

Sexual assault is, unfortunately, common internationally. According to one report, rape has an estimated lifetime prevalence of 19.3% in women in the USA (Breiding et al., 2014), although others have reported higher numbers (Koss, 1993). In Norway, lifetime prevalence has been estimated at 9.4% in women and 1.1% in men (Thoresen and Hjemdal, 2014).

Kelly (1988) has described how sexual assault needs to be recognized in its many forms. According to the US Department of Justice, sexual assault can be defined as “a nonconsensual sexual act” (The United States Department of Justice, n.d.). In this study we defined sexual assault in accordance with Norwegian law as involving situations where the perpetrator has obtained sex with the victim (or obtained that the victim had sex with him/herself or with somebody else), by force or threatening behavior; or having sex with somebody who was not in a position to defend him- or herself (Straffeloven, 2009, paragraph 291). This definition includes situations that were nonconsensual because the victim was incapacitated.

Research has repeatedly reported that sexual assault often has serious detrimental effects on victims' psychological health (Dworkin et al., 2017). Studies of the prevalence of diagnosed Post Traumatic Stress Disorder (PTSD) after sexual assault have reported a rate of 15.25% for a sample of Chinese women (Sui et al., 2014) and 39% in a Swedish sample (Tiihonen Möller et al., 2014). In US samples, Dworkin et al. (2019) cited research that within the first month post-assault, 67–94% of survivors showed sufficient symptoms to warrant a diagnosis of PTSD (if such symptoms prevail beyond the first month, this is a criterion for diagnosing PTSD). Characterological self-blame, which indicates that a negative view of oneself has been adopted, is common after sexual assault, and such beliefs about oneself and one's identity may have a central role in understanding the negative effects of sexual assault. A rigorous study with sexually assaulted female adolescents showed that the reduction of negative posttraumatic cognitions such as “I'm incompetent” and “the world is completely dangerous, no one can be trusted” mediated a reduction in PTSD symptoms and depression after prolonged exposure therapy (McLean et al., 2015). This suggests that self-blaming cognitions may play a role in explaining the development of PTSD. Internalized identities may also change behavior. For example, Niehaus et al. (2010) reported that identities (“self-schemas”) developed through childhood sexual assault may explain later risky sexual behavior.

Trauma, such as sexual assault, has been described as shattering ideas of oneself, others, and the world and necessitating a renegotiation of one's identity (Janoff-Bulman, 1992). Assault may be so unexpected and unbelievable that making meaning after the event may be fraught with difficulties, an “identity shock” (Muldoon et al., 2016). Duma et al. (2007) interviewed female survivors living in South Africa and reported that their goal was to ultimately “return to self.” These women described a series of (conscious and subconscious) ways in which they worked to “return to self” as defined by them.

According to self-schema theories (Regehr et al., 1999), our understandings of ourselves are formed in relation to others and can be about different aspects of oneself, for example one's “sexual self.” Andersen and Cyranowski (1994) defined sexual self-schemas as “cognitive generalizations about sexual aspects of oneself” that are “derived from past experience, manifest in current experience, influential in the processing of sexually relevant social information, and guide sexual behavior” (p. 1079). Self-schemas, much like Bowlby's “working model of the self,” refer to internalized ways of understanding oneself, including one's thoughts, feelings, and behaviors. Self-schemas may influence later coping behavior. For example, a qualitative study reported that self-schemas developed in interactions in childhood seemed to influence these individuals' coping after sexual assault in adulthood (Regehr et al., 1999).

Moreover, interactions with others who learn about the assault may influence how survivors perceive themselves and thereby the process of recovery—how they “return to the self.” Holstein and Miller (1990) argued that individuals who have experienced sexual assault may easily “lose control over their self-definition” (p. 119). Disclosure recipients may have strong expectations, emotions, and opinions about the assault and the assaulted individual. The responses of disclosure recipients are related to the risk of subsequent ill health such as PTSD (e.g., Ullman and Peter-Hagene, 2014). Research on the implications of social reactions to disclosure of sexual assault for recovery has described which responses commonly are helpful and unhelpful. For example, disbelieving or blaming the assaulted individual has been reported to be a common unhelpful disclosure response (e.g., Filipas and Ullman, 2001). Which identities are developed after trauma is important, as identities used to describe oneself after a trauma may shape recovery as well as mental distress (Boyle, 2017). For example, negatively defined self-representations may prevent survivors from defining their experience as an assault and disclosing it to others (Halvorsen et al., 2020).

McKenzie-Mohr and Lafrance (2011) and McKenzie-Mohr (2014) explored available narratives after sexual assault and described two “master-narratives.” Both master-narratives are binary in that they each describe two identity poles that seem incompatible. The “negate or blame” narrative conceptualizes assault as “just sex” (and thereby not an assault) *or* blames the assaulted individual for the assault. The other master narrative is the “trauma of rape” narrative, where assaulted individuals are understood as traumatized and not to blame for the sexual assault. This narrative offers an alternative to the negate or blame narrative but may also be seen to imply that assaulted individuals are somewhat damaged, weak, and always in need of professional help. These two narratives may place assaulted individuals in a dilemma: as either being a credible, helpless, and weak victim, *or* a blamable active partner who called the assault upon themselves. McKenzie-Mohr and Lafrance (2011) introduced the concept of “tightrope talk” to refer to a survivor's efforts to cope with this dilemma. This involves finding ways to define themselves without being blamed for the assault, but also without being perceived as passive and damaged victims. Women in McKenzie-Mohr's (2014) study showed that it was possible to position themselves as both active agents in their lives, and as victims.

Resent research suggests that rejecting binary identities such as those described by McKenzie-Mohr and Lafrance (2011) and McKenzie-Mohr (2014), may have become increasingly common. A recent study among American college students reported that as many as 44% of the participants identified themselves as *both* victims and survivors (Boyle and Rogers, 2020). Moreover, blaming or disbelieving the victim seems to have become less politically acceptable in many societies, including in Norway, which has been described as a “feminine” culture (Hofstede, 2000). According to studies among American college students and community residents, positive, non-blaming, and believing responses to rape disclosure may be the most common, at least among informal support providers (Ahrens et al., 2007; Dworkin and Allen, 2016). However, most studies on disclosure responses seem to have been conducted with college samples (e.g., Orchowski et al., 2013) or community samples (e.g., Ullman, 2010). Studying a help-seeking sample may add to the literature by expanding our knowledge of survivors' post-assault experiences.

Given the interpersonal process of meaning-making and identity formation, it is interesting to learn more about how survivors of sexual assault experience the process of negotiating an acceptable identity in non-blaming and believing interactions with others. We explored this by interviewing a sample of help-seeking survivors of sexual assault about their experiences with non-blaming post-assault interactions with others that affected them either positively or negatively.

## Method

Our aim was to explore first-person accounts of women's experiences of concrete dyadic conversations after a sexual assault, and consider the research question: “How do survivors work toward developing acceptable identities after sexual assault, in interactions with others that are not blatantly blaming or disbelieving?” We used a reflexive thematic approach to analyze transcripts from individual, semi-structured interviews (Braun and Clarke, 2006, 2019, 2020, 2021a,b).

Braun and Clarke (2021b) highlight the need to be transparent about one's paradigmatic and epistemological assumptions. They note that there are several types of thematic analyses, and that these are not atheoretical and can rest on different epistemological and paradigmatic assumptions. They describe three main types of thematic approaches. These range from what they call “coding reliability approaches” which are often post-positivist and aim to secure unbiased and objective coding, to a reflexive thematic approach, in which the researcher's subjectivity is seen as an analytic *resource* (rather than a problem), and coding is “unstructured and organic, with the potential for codes to evolve to capture the researcher's deepening understanding of the data” (Braun and Clarke, 2021b, p. 39). Each of these types of thematic analysis uses different quality-criteria. We aim to meet quality criteria specified by the latter “reflexive thematic approach” (Clarke and Braun, 2019). We focus on being reflexive and transparent about our (subjective) input when construing the findings, to clearly specify the epistemological assumptions and guiding theoretical frameworks for the analysis (so that readers know where we are “coming from”), and to fully develop the themes. We place ourselves slightly more toward the critical realist end of the interpretivist/constructionist—essentialist/realist dimension. We agree with Kvale (2007) that the way interviews are conducted is important. The study interviewers aimed to understand the participant's thoughts and feelings, and to check their own understandings during the interview itself. This was done by rephrasing the words of the interviewee to check the interviewer's interpretations of what they heard, and to be sure to do so before the audio recorder was switched off. To further reduce the risk that interviewers and participants were talking past each other, interviewers asked for specific examples whenever participants used abstract descriptions (Haavind, 1987). Thoughts, feelings, and behavior were sometimes probed to obtain a fuller picture of the participant's experiences if they spontaneously mentioned only one of these aspects. Since we (subjectively) interpret participant descriptions beyond what participants explicitly expressed, we continually reflected upon our contributions in interpreting and developing the findings (reflexivity).

### Procedure

Participants were recruited over a period of 2 years from a clinic that offers psychological therapy for trauma-related crises in a medium-size town in Norway. The second author works at this clinic as a facilitator of groups for women who have been sexually assaulted. These groups aim to provide a place for survivors to share their experiences with other survivors. Survivors are mostly referred to the clinic from the community rape reception center, their physicians, or other psychologists at the center. The second author sent a description of the study [as approved by Regional Committees for Medical and Health Research Ethics (2017/2098/REK vest)], to former and present group therapy participants. This letter informed patients about the procedures and their rights as research participants.

Inclusion criteria for participants were being adult (>18 years) and having been sexually assaulted as defined by Norwegian law, as described in the introduction (Straffeloven, 2009, paragraph 291). Since the exclusion criteria for group therapy at the clinic were being psychotic or actively struggling with substance abuse, and having experienced incest as a child, these criteria were also the exclusion criteria for this study. Contact information for participants who wished to take part (approximately half of those who received information) was forwarded to one of five trained graduate psychology students carrying out the interviews. Interviewers were in their last year of studying psychology at the local university, and had training in clinical work as well as conducting qualitative research.

### Subjects

All participants had sought professional help after the assault, and they were waiting for, or had completed, a group-based intervention at the site of recruitment for this study. One of the women who agreed to take part could not be reached, leaving 15 participants. Consistent with the demographics in Norway, all but one of the participants were White/Caucasian (*n* = 14). Most of the participants had participated in six group discussions with other survivors, and a few had also had 1-3 individual therapy sessions. Participants were 20-45 years old (mean: 28.5 years). At the time of the assault, they had been 17–44 years old (mean: 25 years). In all cases there was one perpetrator. One participant had been assaulted twice, by two different men. The assault had happened less than a year ago for seven participants, between 1 and 3 years ago for five participants, and more than 3 years ago for three participants.

### Interviews

All interviews were conducted in localities at the crisis center, since we regarded this as a familiar and safe place for participants. The semi-structured interviews lasted from 40 min (one interview) to 2.5 h (two interviews), and were audio recorded. Although we were interested in how the interactions with others influenced their experienced process of developing a post-assault identity or self-understanding, we did not ask directly about this. We believe that the ways identities are formed through interactions are most probably implicit and “lived” rather than explicit and conscious, so that we would gain access to data that made it possible to explore our research question by probing for specific examples from post-assault interactions with others. This is reflected in the construction of the interview guide (please see Supplementary Material). During interviews, participants were asked to share their experiences with regard to a conversation, good or bad, that they remembered having with someone about the assault, and that had affected them in some way. When recounting specific interactions during a conversation, they were asked how that interaction had affected them, and sometimes what they thought the disclosure recipient was thinking. If they described a bad conversation, they were next asked about a good conversation, and vice versa. Finally, they were asked if there were any other conversations they would like to include. Participants were not asked about details around the assault itself, for ethical reasons. At the end of the interviews many participants spontaneously reported that the interview had been a good experience, and commented that research was important, since it might help others who had been sexually assaulted and increase the competence of professionals.

### Analytic and Reflexive Processes

Interviews were transcribed verbatim and analyzed within the framework of reflexive thematic analysis (Braun and Clarke, 2006, 2019). We looked first for semantic and then latent themes as our understanding of the interviews developed. The first and last author were responsible for the development of the analytic process. The first author initiated the project and supervised the students carrying out the interviews. Throughout the project she was very conscious of trying to avoid that the findings presented would contribute to limit the available identities and positions for survivors of sexual assault. She also has a special interest in the dilemmas that assault victims may experience in interactions with others. The second author works clinically with survivors of sexual assault. She recruited the participants, gave advice about sensitive interviewing and commented on the interpretations of the results. The third author was not involved in the project development, but has had an active role throughout the process of analysis together with the first author. She has worked clinically with survivors of complex trauma for several years and has undertaken several research projects exploring the first-person perspective of disclosure of child sexual abuse, help-seeking following complex trauma, experience of trauma treatment and processes of recovery from complex trauma. This experience and interest have informed her position in the analysis, although she has worked to stay open and in tune with the experiences shared by the participants. The slightly diverging positions of the first and last authors were used actively throughout the analytic process, helping to see how our positions influenced our understanding of the data, and to broaden and develop the analysis further than we would have been able to on our own.

Analyses progressed from being mostly bottom-up to becoming more concept-driven as themes were developed. The analysis started with the first and third author reading the transcripts several times to familiarize themselves with the data. The first author then coded the interviews, marking and naming all text relevant to the analytical focus (bottom up). At this point in time, she was looking for any instances relevant to the broad question: “What characterized these interactions as they were experienced by the assaulted?” Based on this analysis, 19 broad codes were constructed, representing tensions between different positions and coping strategies. Examples of these codes were: “normal and understandable vs. different,” “closeness vs. distance,” and “strong person vs. weak victim.” She alternated in this process between using NVivo12 as technical support and writing and developing an analysis in Microsoft Word. At this stage, the NVivo file and a Word document outlining a tentative thematic structure was sent the third author. As the codes gained more examples from the interviews, and through discussions between the first and last authors, the themes started to take form, and it became clear to us how participants navigated the tension between different needs and ways of coping while working to rebuild their post-assault identity. The first rough draft of the results section was then read by the second author, who gave feedback from her perspective. The first and last author then had regular meetings, worked with the texts together and sequentially, and went back to the transcripts to check that the interpretation and evolving thematic structure was still closely linked to the participant's expressed experiences and examples. During this process, the thematic structure was modified several times, including collapsing tentative themes into broader themes, finally resulting in the presented thematic structure of four themes. Differences in the initial understanding of the data material and emerging thematic structure were resolved through discussion, reflection, and clarification of our own positions, and how they influenced our understanding.

### Ethical Considerations

The study was approved by the Norwegian Regional Committees for Medical and Health Research Ethics (2017/2098/REK vest), so it complies with the Declaration of Helsinki. The participants in the study are part of a potential vulnerable group, as they are survivors of sexual assault who have sought help. Moreover, the dual roles of the second author call for high degrees of reflexivity to ensure the fulfillment of important ethical principles, such as voluntary participation and informed consent. Although the second author's position facilitated engagement with the field and access to the phenomena under study and potentially made participants feel safer, we had to find ways to avoid a situation in which her dual roles might have led to pressure to participate or social pleasing accounts during interviews. Yet it is also important to find good ways to facilitate research participation among vulnerable groups, to ensure that their perspectives are heard. To safeguard the fulfillment of key research ethical principles, while allowing participants to share their perspectives, we clearly communicated all relevant aspects of the study in a written letter before participants initiated their participation. Interviews were conducted by students with no previous or existing relationships with the participants, and the focus in the interviews was neither on experiences from therapy nor on experiences of the assault. Moreover, no reminders were sent out, to minimize the risk of participants feeling pressured to participate. Our assessment is that these measures were sufficient to safeguard the well-being of the participants and the principles of voluntary participation and informed consent.

## Results

In the following, we call the person who interacts with the participant a disclosure recipient, although interactions described might not be the first time the other learns about the assault. We use the concept “identity” to mean both the image subjects may have of themselves based on their self-representation and the way others see them, their role, or their social identity. Information from the context of the citation in the interview is provided in square brackets when needed to make the citations understandable. Pauses are indicated by ellipses, and omitted parts of the citations are indicated by ellipses in brackets: (...).

Participants most often described discussions with friends, families, colleagues, acquaintances, and professionals at the emergency room. Only rarely did they describe discussions during their therapy in particular, although some underscored the benefits of being in group therapy with others who had experienced sexual assault, as this helped normalize their own reaction in their minds.

We organized the material in four themes that together describe processes at work when negotiating an acceptable identity through post-assault interactions with others. The first theme, *Navigating between others' story and one's own*, encompassed the process of finding words to describe what study participants had endured and the interplay between receiving help to understand what had happened to them and their own role in the assault through interactions with others, and the risk of being defined and trapped by others' stories of the assault. The second theme, *Realizing the seriousness of the assault without drowning in the upset of others*, involved positive experiences of being reminded of the seriousness of the assault through interactions with others, as well as negative experiences in being engulfed in others' strong upset about the assault in ways that made no room for their own feelings. The third theme, *Finding a place between too much closeness and too much distance*, involved the tension between positive and negative aspects of others' bid for closeness after the assault, and processes of bridging previous and current roles and needs in relation to closeness in relationships with others. The fourth theme, *Being more than a victim: letting things be normal too*, involved the tension between positive and negative experiences with being seen as a victim by others.

### Navigating Between an Others' Story and One's Own

Participants described a search for words to describe themselves and their feelings that could be shared and validated, especially shortly after the assault but also some time afterwards. They could be shocked and confused, wondering if their words were understandable at all:

And I think I was so distant . . . [even now] often when I talk, it makes me say things that don't even make total sense for me. Or I feel so distant that sometimes when I talk it's like I hear myself talking in the middle of a sentence, and I've almost forgotten what I was talking about. And things go sort of on autopilot and then one becomes a bit . . . eh . . . I become a bit, eh . . . surprised that people have been able to understand what I'm saying at all. (Elizabeth)

In this state, the others' perspective on what had happened would often make a strong imprint:

And that's something I don't think people always realize, how much the words they say, how much those words are analyzed up and down and forward and backwards. (Isabel)

The participants struggled to determine their role in the assault. The input of others could be essential to avoid unfair self-blame and rumination. For example, Elizabeth noted that if she had been alone when trying to determine her role in the assault this might have increased the risk that she would blame herself and start to believe that others might also blame her:

So it was good to . . . in a way receive confirmation that what I say makes sense, and confirms what I feel, is correct. This [the assault] has actually, happened—this is not something in your head.

The influence of others could bring order rather than chaos to a participant's understanding of the event, and counteract identification with being confused and crazy. Melody said talking to a professional helped her realize that she hadn't “turned totally crazy”:

Many times, since it happened, I've felt that I've sort of lost a bit of confidence, started to doubt myself a bit. Especially after I reported it . . . when you're asked to tell about it repeatedly and may start to become a bit unsure and doubtful, “Was it . . . really like that?” Was that exactly what happened? And . . . so many thoughts about that, that it . . . It helps when somebody just listens to what you say and tells you it makes sense. (*Yes*) in a way. It validates that you haven't turned totally crazy.

However, input from others could also be experienced as unhelpful. For example, Ann felt uneasy about comments she received at the rape reception center shortly after the assault. When a nurse commented, “Oh, I know this must be awkward for you,” Ann felt like replying, “You don't know *anything* about what I'm feeling!” Isabel described a struggle with finding words, and frustration when others put words in her mouth:

Because what one is trying to do, one is trying to express something that one actually doesn't have words to talk about. And then, somebody takes over and does it for you, and—for me, at least—that made me angry [short laugh].

Other people's assumptions about the survivor could hurt, even if not directly unfriendly or totally incorrect, and rejecting their definitions could be difficult. For example, a friend of Isabel's made a comment that Isabel had “a strong need for control” after the assault. Isabel felt this was wrong since she felt it would be more correct to say that she had a strong need for security. However, she did not explicitly reject the definition there and then: “I responded by shutting up and thinking to myself, ‘Yes, I kind of made a fool of myself there’; I thought I could open up but she defined me.”

For some survivors, expressing one's perspective could be a protection against other people's stories:

So it's in a way in order to protect myself, the story is mine and no one else's in a way. That's why I don't want people to interpret that story in all kinds of directions. (. . .) I strongly need to feel in control of the story, I need to feel that it's me [who tells it], sort of, it's my version, that is communicated. (. . .) So, eh, that might be the most important way in which I've changed. (Isabel)

When Kate was asked what advice she would give to disclosure recipients, she responded, “Don't assume.” She explained:

Some people have gone, like, “Well, I guess you feel guilty for what happened.” And it's been, like, people have sort of put words to what I'm [supposedly] feeling, without me having the chance to say it myself.

We suggest this shows that accepting others' input could be helpful for participants in their construction of their own story of what had happened to them, however, as we have seen, the input of others could also disturb the process of regaining one's footing and trust in one's own self-definition.

### Realizing the Seriousness of the Assault Without Drowning in the Upset of Others

This second theme revolved around the participants' process of realizing the emotional impact of the assault and sorting out their own feelings in interactions with others. Disclosure recipients' communications that the assault was, in their eyes, serious, could be helpful. Participants described that others' reminders of the seriousness of the assault helped them realize how severely the assault had affected them. However, others' input about the seriousness of the assault could also make the survivor feel uneasy. Others' communication of strong emotions when learning about the assault could also feel wrong to the participants, as if others had taken the right to define which feelings the assault now called for.

The positive aspects of being taken seriously when talking about the assault were evident. Other people could remind participants that they were not as untouched by the assault as they thought. This could counteract tendencies to attribute their experienced difficulties as an expression of their own overreacting or being lazy. For example, Grace received help in accepting herself as deserving to be on sick leave:

I tended to struggle with a very bad conscience, feeling guilty about being on sick leave. But [when talking to others] one starts to understand [and accept] that one isn't as capable [after an assault] as one should have wished.

Similarly, when asked how friends had helped her after the assault, Kate said that they helped her by reminding her that the assault might explain some of the difficulties she subsequently experienced at school:

If I compare my concentration and school achievements before and after the assault, I see that they have become very different. I tend to become very disappointed if I'm not able to achieve things [in general]. And in those cases, it helps that others... If I complain to a friend that I got a bad grade, he can remind me, “Well of course, just relax, it's not important,” and in that manner be very helpful, helping me to see that there is an association [between having experienced the assault and getting worse grades]. That the reason is not just that I..., it's not just me, there is *another* explanation.

However, others' taking the assault very seriously and expressing this in the form of strong emotions, could also be negative. Their upset could define the survivor as damaged and close to drowning, and was likely to disturb the participants in their search for their own sense of how the assault had affected them. The survivors lost the position of defining and expressing feelings about how the assault had affected them. For example, Elizabeth said that she had preferred that her friend “just talked normally” rather than showing strong upset and anger when Elizabeth told her about the assault. She commented: “This [the assault] is sort of my thing and not . . . [hers]. She didn't, kind of, have the right to . . . eh . . . get angry about it. I don't know, it's kind of strange [that I feel this way].”

Kate was relieved that she could talk with her sister about the assault in a slightly humorous and “matter-of-fact” way. This was in line with her sense of being a person who preferred to talk about things more matter-of-factly and with less explicit emotionality: “I'm a big fan of just doing it in a simple way and not taking it too seriously. Because everybody knows that this is serious, so one doesn't need to make it even worse.”

When others became so upset and angry that they expressed a need for violent retribution against the perpetrator, this could deprive the assaulted of the role of defining which actions were wanted and needed. For example, Fiona noticed that her boyfriend was highly distressed about the assault and had said that he would have killed the perpetrator if he had him there. This made her downplay the seriousness of the assault when talking with him, which she realized caused problems in their relationship. At other times, peoples' upset expressed helplessness. This could make the participants feel obliged to help, which deprived them of the room to find their way forward and receive the support needed to do this. As noted by Grace, the survivor might not be and not feel like “the one who is in the right position [to help distressed relatives].”

We interpret these stories as implying that participants needed to find a way forward bearing their identity as survivors without being swept off their feet by the force of others' reactions. We believe survivors might feel pulled in different directions, wishing that everything would go back to normal and defining themselves as a person who could be expected to quickly recover, to being overwhelmed by the experiences and the reactions of others, feeling like someone whose fate was so overwhelming, unfathomable, and unmanageable that it necessitated expressions of strong feelings of powerlessness, helplessness, and rage. While interactions with others helped the participants get in touch with the seriousness of the assault and sort out feelings of guilt and shame, the reactions of others could also make it more difficult for participants to sort out their own feelings and reactions to the assault, and were thus a threat to the process of establishing an acceptable identity following sexual assault.

### Finding a Place Between Too Much Closeness and Too Much Distance

This theme revolved around taking a stance on how much emotional and physical closeness one wanted, following the assault, and how this was negotiated in interactions with others. On the one hand closeness was clearly positive and needed. On the other hand, closeness was troublesome. The survivor needed to retain her identity as an agent who could accept as well as reject closeness in the relationship at any one time.

Participants described how closeness could be positive and make them feel cared for. Other people checking on them and asking about the assault in a kind and concerned way could be helpful. For example, Helen wanted others who knew about the assault to check on her now and then: “I know it's hard [for others] to . . . know what to say, but just to know that they are there for you [is good].”

Emotional closeness could also be experienced negatively. Being asked about one's current state (“how are you really feeling”) reminded survivors that they were a person who had been assaulted and forced them to move closer to their own feelings. When others insisted on talking about the assault, this could prevent participants from attending to other things and developing “new memories” rather than the ones connected to the assault: “I just want to have my memories not connected to this particular thing, you know, just to have new memories. And to . . . That's why I don't like to think about this.” (Helen). Isabel noted that when someone mentioned the assault she sometimes felt small, and this would remind her of the situation where she was assaulted, which would trigger the whole sequence of feelings that she felt at the time: “I felt very small, and when I detect just a tiny bit of that feeling [of being small when talking to somebody], the whole sequence of feelings, like guilt and shame and all, return.”

Isabel described the duality of both wanting others to understand that she was struggling with having been assaulted on the one hand, and not wanting to talk about the assault on the other:

So while at the same time I want people to understand why I'm a bit zoned out, or why I can be strange, (. . .) I am commonly outgoing and now can be a bit reserved, . . . I would like people to know why. But at the same time, I don't want to tell. I have a kind of duality inside: I want people to know [about the assault and how I feel], while at the same time I don't.

Questions from others about how the participant was “really” feeling could feel intrusive, as if the person asking was implying that they had a right to know, or that the assaulted was in denial or covering things up, and needed to talk it all out.

If the relationship is, like, “How do you really feel, now?” it can be a bit intrusive. . . . It's like pressing the person into a corner. . . . You don't get the chance to escape if you don't want to [talk], you are sort of forced to take that serious talk. Those kinds of situations are very difficult. (. . .) [I feel like saying] “Don't remind me, don't pull me into that shit.” (Grace)

Learning about the assault naturally drew many family members closer, with the intention of monitoring the participant's mood and general well-being. This was often greatly appreciated, however, participants also needed some emotional and physical space. For example, Helen's mother lived abroad, so writing or calling was her form of communication:

But since then [since the assault] she was just [phoning or writing] every single day, or twice a day. And then it was just too much. It came to a point that I had to say to her: stop. Because every time I'm gonna look on the phone I'm gonna remember what happened. And it just takes me back, every single time. I said, “I'd rather, see, you to call me, I don't know, once a week?” than to keep doing that. I mean it was just her way to [show she cared].

Identifying oneself as a person who needed more distance could create feelings of guilt. Ann described keeping herself geographically separate from her parents for a period after the assault, in order not to have to deal with their sadness. She tearfully admitted to feeling guilty about not letting her parents close to her and not letting them help her in the manner that they felt best, and knew that the resulting physical distance between them frustrated her parents:

Because they felt they were not able to help me. I didn't want them to visit me, and I didn't want to come home until Easter. (Yes) I couldn't endure having them around and I didn't want them to analyze me, when this meant that I needed to see how sad they were.

Closeness could also be communicated physically. Helen expressed gratefulness that her partner understood “that he couldn't just hug me or something.” However, it had been hard for her to tell him that she needed space, to say, “Don't touch me, leave me alone.” This could evoke guilt on her part, for keeping him away.

The participant's wishes to adjust the amount of geographical, physical or emotional closeness, combined with a real appreciation of the other person, could be difficult for both participants and others to fathom. Elizabeth struggled with an experience of herself as curt and ungrateful when she felt a need for distance:

I become a bit curt. Not that I want to be curt, but it's perhaps just a bit, “Augh, keep away from me,” in a way. But [this loved one] has been extremely kind too. A bit of both. She has really been there for me and fixed very many practical things on the one hand. At the same time, her worry for me is a bit strenuous. And, ehh. Cause she's the one, I guess, who is most often asking me, “How are you doing?” and such. And she can suddenly become... it can become a bit exhausting. (...) Perhaps it's something about just letting the [assaulted] person herself have control.

The unambiguous support and care of others, expressed through emotional and physical closeness, was needed, then, yet, the participants found their needs and tolerance of closeness being changed by the assault. Finding a position between closeness and space after the assault, and taking a stance on when one wanted to talk about the assault and when not, could therefore be challenging in post-assault interactions. The participants needed to find a way to communicate their current need for both closeness and distance, without alienating others whom they loved and needed.

### Being More Than a Victim: Letting Things Be Normal Too

Clearly, the participants realized that they had been hurt, and appreciated the care and concern of others, however, at the same time they didn't seem to want the assault to dictate how others treated and saw them. They wanted those parts of themselves that they called “normal” to be recognized. Rather than being treated only as fragile and totally changed, participants struggled to retain an identity that allowed them to be seen more as they were before, in interactions with others. For example, Isabel explained:

Well, maybe what has been most challenging for me [after the assault] (...) [is my fear that] people will think, “Wow, she is about to panic, and then I need to take care of her.” And I don't like that, because then I'll be a victim again. (...) In a way, everybody has an opinion. I don't want pity; instead I want, “This is going to be fine, we will manage this, what can I do for you?”. I don't want responses like, “Oh my, you must feel terrible.” (*Interviewer: How does that pity affect you?*) No, I think, it may just... it doesn't help me get free from it.

Being treated “as normal” implied being assigned some of the same characteristics as before the assault, and the same “normal” identity as others. For example, Kate wanted her friends to treat her as they would have done normally before the assault:

“I have a strong need for them to treat me as when things are normal [as before the assault] (*mm*) I don't, know [how to put it], I understand, I appreciate that people show compassion and sympathy, but I don't want it all the time. It becomes a repeated reminder of why they are doing that [being kind] to me.”

Being seen and treated as normal involved rejecting negative, but also unrealistically positive, identities. Nora described an example of how an unrealistically positive identity could feel like a cover-up for other people actually seeing her as a victim whose self-confidence needed to be artificially bolstered:

[*messages*] may tick in on my phone: “Oh, you are so tough and you are so strong,” and then, “heart, heart, heart” all the time. That [those messages] makes me feel very, actually, a bit worthy of pity. . . . It's like [they are saying], “Poor you, you are only pretending to be tough.”. . . Because it's so easy to receive that kind of comment, “You are strong” or “How tough and strong you are.” One might not do anything that's strong or tough, but the list [like in height jumping] is put so low for you [the criteria of being strong is so easy to fill], so then it's very little that is required for me to manage to reach it. (. . .) In those cases you feel that things are not normal, that they [friends] do not perceive you in the same way as before.

Melody explained that she preferred not to let the assault ruin her identity as an adult with plans for the future:

*(Interviewer: What would you say has been important for you after the assault?*) Hmm. I guess just looking to the future, . . . to continue [my life]. That has been very important for me. I don't want to fall into a victim role, because I don't feel like a victim. So yes, I guess that's what's been important for me, really. Looking forward, being able to think about the future. . . . I won't allow this to ruin me. In any way.

She also wanted to remain a person who was not only not fragile but also one to whom others could come for help:

And I don't want people who know, to go around and think about it all the time. [I don't want] for it to be something that worries them. So it's a bit like, “I want us to just ‘park’ it a little.” (...) And that they can come to me with their own, all kinds of, problems; and that they don't have to be considerate of me.

She gave an example of one friend who let her be strong in relationship with him:

He's more at ease with coming with his things to me. If something is the matter [in his life]. It's more like we are friends, that we both can actually tell each other about things that are not so good, too.

She valued being seen by others as a friend who could still appreciate humor: “He had bought some grapes, so he offered me a grape and said, ‘This is not a sympathy grape but a between-friends grape,’ and then he simply followed me home.” Retaining an identity that expanded that of a mere survivor also involved finding a balance between making one's own decisions and being guided or pushed by others. Protection and guidance could feel right, and being pushed to seek help or join the company of others could be precisely what was needed. Helen said of her boyfriend, “He suggested what I needed to do, and he was there for me.” Beth felt grateful for her friends pushing her to “keep doing things” (e.g., seeing films with them or talking with them):

It's been extremely important for me, that my friends have been so supportive and good to me through it all. And . . . they have pushed me to keep doing things, while at the same time, when they have sensed that I'm not ready, they have backed off.

However, being given an identity of needing other people's “pushing” and opinions could be restricting. Grace recalled a phone call from a close friend who wanted her to talk on the phone with a woman who had herself experienced assault, because “this could be good for her.” This was shortly after the assault, when Grace was busy trying to get back to her routines and to continue her professional work. The offer to talk with this woman on the phone came suddenly and without forewarning. Rejecting an identity as being a person who needed therapy evoked feelings of guilt in Grace:

I didn't want that, I didn't want to go to therapy. I didn't need that sympathy. If I ever talk to her again, I have to apologize for my response. But [at that time] I didn't need that kind of help. I absolutely did not want that [kind of] help from anybody, actually.

After the assault Ann intensely disliked being told that she had to report the event to the police, or that she “had to” come dancing, or “had to” watch a certain TV series:

I've become much more sensitive to being pressured. I don't like to be told, “Oh, you just have to come dancing,” because I really don't “have to” anything! Or, “Oh, you just have to see that TV series.” In those cases I just want to show some defiance and say, “I don't have to do *anything*.”

Fiona noted that although she was afraid after the assault, she resented the restrictions that others sometimes put on her freedom of choice. She talked about friends who wanted her to avoid taking the local bus:

I don't want to avoid those things that I used to do. This [the assault] shall not hinder me in doing everything my way. Of course, the thoughts [about being in danger] come creeping in some situations. But it probably won't be better if I avoid them. If one avoids them it will be much more difficult to resume the usual things.

Helen explained that her mother did not want her to go anywhere without the company of her boyfriend, but felt reluctant to let the assault affect her life in that way:

And one day I was talking with my mum, and she told me—because I go hiking a lot—and she told me, yeah, “You need to make sure he's always gonna be with you.” And then I felt so trapped. But I don't want that, I don't want that one person [the perpetrator] can influence my life in that, in that kind of way, so that I become so afraid that I cannot go anywhere. Ahh.

In summary, the participants described how in interactions with others they balanced the need to be recognized as a person who needs support and “pushing,” on the one hand, and the need to be someone who could be relied upon to decide for herself how to continue her life.

## Discussion

We suggest that the presented findings broaden our knowledge by presenting an in-depth analysis of post-assault identity processes in interactions with others. We will discuss these findings in regard to the help giver/help receiver interactions that may characterize post-assault interactions.

In the introduction we suggested that to work toward acceptable identities following sexual assault means to negotiate a way of looking at oneself, and being looked at by others, that is acceptable to oneself and understood, acknowledged, and respected by others. In the following we will argue that seeking an acceptable identity in post-assault interactions involves finding a way to combine a help-receiving (victim) role and an agent role.

The interviews suggest that the conflicts and challenges inherent in establishing an acceptable identity after sexual assault are a result of the combination of having been severely hurt and at the same time wanting to “be oneself,” as before. Sexual assault makes victims painfully aware of their identity as vulnerable to crime and in need of support by others, which may collide with their original identity of being “normal,” safe, and strong. It may seem that only a victim role is available. As described in the introduction, McKenzie-Mohr and Lafrance (2011) and McKenzie-Mohr (2014) have argued that the availability of only a few, contradictory and restrictive, narratives may place assaulted individuals in a tense dilemma. The dilemma is that neither “pole” of the binary identities represents an acceptable identity. Survivors may need to choose between being assigned an identity as a credible, helpless, and weak victim, *or* being assigned the identity of a blamable active part who had called the assault upon themselves (McKenzie-Mohr and Lafrance, 2011; McKenzie-Mohr, 2014).

Participants in this study were not blamed by others for having caused the assault. In effect, they did not need to escape the “blamable agent” identity (at least not from others, although many had blamed themselves). However, similar to women in McKenzie-Mohr and Lafrance's (2011) writings, they did seem to be aware of the possibility of being trapped in an identity as only a passive victim. Like women in McKenzie-Mohr and Lafrance's (2011) study, survivors seemed to attempt to fight “traps” of being restricted to victim identities with little agency. They expressed wishes and characteristics that did not fit the identity of being only a passive and help-needing victim and instead fought to regain their agency and their footing. Examples in this study were interactions where survivors wanted to define their feelings themselves, wanted to be in charge of expressing the seriousness of the assault rather than be overwhelmed by others' upset, and when they wanted distance rather than closeness and freedom rather than protection. By doing this the participants show how they are actively navigating interactions with others after sexual assault, utilizing available help and support in these interactions to facilitate their processes of identity negotiation, while being aware of potential obstacles interactions with others could pose to these processes. The results in this respect concur with current research from the US showing how many survivors embrace an identity of both a victim and a survivor (Boyle and Rogers, 2020).

Prior quantitative studies have shown that having a sense of agency and influence over the recovery process is crucial for healthy recovery (Ullman et al., 2007; Walsh and Bruce, 2011; Orchowski et al., 2013; Ullman and Peter-Hagene, 2014). A longitudinal study by Frazier (2003) showed that survivors who reported more control over the recovery process consistently reported less distress over time. Being an active agent in the described interactions meant sometimes making choices that might be perceived as avoidance of the victim/help-receiver role. These choices may be in the best interest of the assaulted, given her context (e.g., it may be healthy at times to avoid thinking about the assault, to seek distance and to act “normal”). However, rejecting the identity of a passive and receptive victim could trigger feelings of guilt in participants and might also confuse disclosure recipients. For example, turning down advice and offers of physical or emotional closeness, could feel like being curt, and evoke feelings of guilt. It seemed like it could be challenging to find ways to reject the victim-role without pushing important others away.

Being an agent means being in charge, making decisions, acting. However, after sexual assault the survivor may also need to be an object of others' help, at least intermittently. Matching this, disclosure recipients may themselves want to take on the role of a helper, when they encounter loved ones who are in pain. When disclosure recipients take the active, agent, helper role, the identity “left over” to the assaulted is automatically a recipient of help, a victim. The survivor is constituted as a recipient of help in the interaction, while the other is constituted as a help giver. This is not problematic in itself, as shown in the interviews. There were clearly positive aspects of being a recipient of help after sexual assault, as when the helper is needed to give emotional support and counteract unrealistic self-blame. However, the interviews also made us aware of how being constituted as a help receiver after sexual assault may sometimes be limiting for the survivor. If disclosure recipients and others assume the role as a subject and helper, the survivor is naturally cast in the role as an “acted on” object and help-receiver. This means that the helper is assumed to take charge, becoming an agent and caretaker, so that what might have been a “mutual” relationship prior to the assault (or even a relationship where the survivor was in the helping role) becomes less “mutual.” As with patient–physician roles, which are mostly expected to be subject–object (I–it) relationships in Buber's terminology (Cohn, 2001), disclosure recipient–survivor relationships may suddenly become I–it relationships, where only the helper is subject and agent.

In the present interviews, survivors seemed to navigate toward being seen as a subject (without losing the care of others). We assume that this could sometimes be confusing to both the survivor and others and might cause tension in the relationship (“what *do* you want”?) as well as guilt in the survivor for seeking distance or rejecting talk, advice, and protection. Survivors' rejection of the help-receiving role may deprive the helper of the helping role (“I don't know what she really wants”) and deprive the survivor of being accepted and understood as a help-receiving and help-deserving victim. In the worst case, the survivors' combination of both accepting and rejecting the expected help-receiver role may be pathologized by friends and family, as well as by the survivor herself, making them conclude that the survivor has become damaged or crazy, if not only rejecting and unfriendly.

We suggest that the findings can have both practical and existential implications for how nonprofessionals as well as professionals can assist survivors' work toward regaining their footing and negotiating an acceptable identity after sexual assault. We have suggested above that this study implies that giving care, and receiving it, is needed but also complicated after sexual assault. There is a risk for survivors to feel uncomfortably objectified or “defined” in limiting ways, even when interactions are believing and non-blaming. Professional and nonprofessional helpers might implicitly limit the survivors' freedom to be an agent and a subject (including the freedom to define him- or herself and his or her present needs). Helpers might think that survivors need care, and they *do* need care. But being an agent is also important. Perhaps care is needed in order to be able to be an agent again. In Buber's terminology, both disclosure recipients and survivors, being agents, would characterize I–thou relationships, as these are characterized by “spontaneity, subjectivity, reciprocity, and recognition and acceptance of the unique other” (Cohn, 2001, p. 170), and also freedom to protest and be undefinable and unlabeled. Possibly, having been made an object by the perpetrator, being a subject in subsequent relationships may be particularly important for survivors of sexual assault. This means that it might be even more essential than in other helper and help-receiver interactions to be aware of the (common and often necessary) “help-receiver as object” and “helper as subject” distribution of roles. Helpers might need to combine a sense of being the one who should know and provide what the survivor needs with a realization that they might not at all know, or understand, the survivors' feelings, wishes, and characteristics. The survivors' freedom to be a subject was severely limited during the assault, and survivors' trust in their own agency and their freedom to define who they are needs to be regained in subsequent interactions.

## Limitations and Strengths of the Study

Only half of the women invited to participate by mail responded to the request to participate, and there were no men in this study. The transferability to other contexts, such as communities at war, other cultures, or groups of individuals who do not take on typical gender-related roles, is unclear. Moreover, all participants had sought professional assistance after sexual assault, which may limit the transferability to groups that do not seek help. As mentioned in the introduction, participants might have been more traumatized, but also perhaps more psychology minded (since they sought help from psychologists rather than physicians or religious leaders) and willing to reflect upon the impact of their interactions with others, compared to the population of assaulted women in general. This needs to be kept in mind when gauging the transferability of these results. Our focus in this study was not on interaction during therapy, which would have required another approach. The second author's role in recruiting participants, and the fact that we were all psychologists, might explain why we heard no descriptions of interactions with psychologists, but only interactions with family members, friends, acquaintances, and, in a few instances mentioned by interviewees, also with police, a lawyer, and physicians and nurses at the rape reception.

A limitation of the study is that we had a relatively small sample size of 15, and only conducted one interview with each participant. More participants or several interviews over time might have enabled a deeper understanding of the sensitive topic of sexual assault and the process of negotiating an acceptable identity following an experience such as assault. We believe, however, that the interviews hold sufficient “information power” (relevant information; Malterud et al., 2016) to add to the current literature, and trust that other studies in other samples, as well as the reader's recognition of these topics in their own experiences, may over time show how local or transferable our interpretations of the interviews may be, to other participants and contexts. We agree with Levitt et al. (2017, p. 12) that “Even if the diversity within a study is extremely limited, as in a case study, research can have adequate fidelity by adding a new perspective to the literature.”

## Conclusion

We suggest that even in believing and non-blaming post-assault interactions, navigating toward acceptable identities may mean working one's way back to being recognized as an agent and a subject in interactions with others. This might mean welcoming as well as protesting others' definitions of oneself and one's needs, sometimes also increasing tension in relationships, as well as within the survivors themselves, as they strive to identify their many feelings. We agree with McKenzie-Mohr and Lafrance (2011, McKenzie-Mohr, 2014) and others that the way forward seems to be to find ways to balance between, and combine, the seemingly opposing positions of victim and agent. Although the identity pole of being defined as a “blamable agent” is not as relevant in non-blaming and believing interactions, there is still a requirement for survivors to combine the seemingly opposite roles of being both an “acted upon” object of others' assumptions, care and agency, and a subject and agent who may sometimes protest being given the identity of an acted upon object. We suggest that, perhaps because an assault so harmfully objectifies and dehumanizes survivors, post-assault interaction might be particularly complicated after sexual assault compared with other trauma, even in supportive, caring relationships. Further studies are needed to explore the meanings of having been objectified in a destructive way by sexual assault, and subsequently being an “acted on” object in post-assault interactions with believing and non-blaming others.

## Data Availability Statement

The datasets presented in this article are not readily available because we did not ask for approval from research participants to share the data collected, due to the nature of these interviews. Any questions about the dataset can be directed to ingrid.dundas@uib.no.

## Ethics Statement

The studies involving human participants were reviewed and approved by the Norwegian Regional Committees for Medical and Health Research Ethics (2017/2098/REK vest). The patients/participants provided their written informed consent to participate in this study.

## Author Contributions

ID contributed to the design and implementation of the research, analysis of the results, and writing of the manuscript. EM contributed to the implementation of the research and analysis of the results. SHS contributed to the analysis of the results and writing of the manuscript. All authors contributed to the article and approved the submitted version.

## Conflict of Interest

The authors declare that the research was conducted in the absence of any commercial or financial relationships that could be construed as a potential conflict of interest.
