# Supplementary material for: Finding One's Footing When Everyone Has an Opinion. Negotiating an Acceptable Identity After Sexual Assault
Source: Front Psychol. 2021 Jul 1;12:649530. doi: 10.3389/fpsyg.2021.649530 (PMC8281139; doi:10.3389/fpsyg.2021.649530)
Supplement: Supplementary file 1 [file Data_Sheet_1.PDF]

Interview-guide

Thank you for coming.

1. May I ask your age?

2. People differ in which words they like us to use regarding sexual assault, some prefer to use the word sexual assault while others prefer to use the word rape, - what term would you prefer that we use?

…

3. How are you today?

4. What thoughts have you had regarding coming here today?

5. How long is it since the sexual assault?

6. How have you been since it happened?

7. What has been important for you in the time after the sexual assault?

…

8. And now I would like to ask you how it’s been to speak with others about the sexual assault. So: I wonder if you can tell me about a conversation that made an impression on you, either positive or negative, where you spoke with someone about what had happened to you.

Probes, if necessary:

a. Who was this?

b. How long after the sexual assault was this?

c. What do you think he/she thought/felt/meant about what you told them.

i. How did you notice that (that they felt, meant, thought this)?

d. What did you think, feel, about what they said? What did you do?

e. Have noticed any changes in how they relate to you, or in their behavior towards you, after that discussion?

f. What do you think all of this (with reference to what the respondent has described regarding interactions) has meant to in the aftermath? (you might clarify by adding: has had an impact on you in the aftermath)

Repeat question 8 in order to explore more of the discussions considered important by the respondent (both good and bad experiences)

…..

9. What feeling do you have regarding how you’ve been met by others?

10. How do you think these reactions had affected you and your coping with the sexual assault?

11. If you were to give any advice to (the police, friends, boyfriend/girlfriend) that could be to any help for others who has experienced a sexual assault, what would that advice be?

12. Are there other things you would like to share about your experiences, anything else that you feel are important?
